# [3-Chloro-*N*′-(2-oxidonaphthalen-1-yl­methylidene)benzohydrazidato]methanol(methanolato)oxidovanadium(V)

**DOI:** 10.1107/S1600536811032703

**Published:** 2011-08-17

**Authors:** Fu-Ming Wang

**Affiliations:** a Department of Chemistry, Dezhou University, Dezhou Shandong 253023, People’s Republic of China

## Abstract

In the title complex, [V(C_18_H_11_ClN_2_O_2_)(CH_3_O)O(CH_3_OH)], the V^V^ ion is coordinated by a tridendate 3-chloro-*N*′-(2-oxidonaphthalen-1-ylmethylidene)benzohydrazidate ligand, one oxido ligand and by O atoms from a methanol and a methoxide ligand, forming a distorted octa­hedral geometry. The dihedral angle between the benzene ring and the naphthyl­ene ring system is 6.4 (3)°. The deviation of the V^V^ ion from the plane defined by the three donor atoms of the tridentate ligand and the meth­oxy O atom towards the oxido O atom is 0.323 (2) Å. In the crystal, pairs of inter­molecular O—H⋯N hydrogen bonds form centrosymmetric dimers.

## Related literature

For background to hydrazones and their complexes, see: Seena *et al.* (2008 ▶); Bastos *et al.* (2008 ▶); Sarkar & Pal (2008 ▶); Nica *et al.* (2007 ▶). For the structure of a related oxidovanadium complex derived from *N′*-(5-bromo-2-hy­droxy­benzyl­idene)-2-chloro­benzohydrazide, see: Wang (2011 ▶). For other related oxidovanadium(V) complexes, see: Kurup *et al.* (2010 ▶); Rajak *et al.* (2000 ▶); Grüning *et al.* (1999 ▶); Mondal *et al.* (2009 ▶).
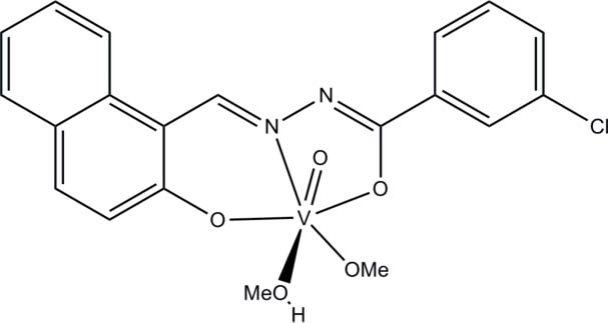


## Experimental

### 

#### Crystal data

[V(C_18_H_11_ClN_2_O_2_)(CH_3_O)O(CH_4_O)]
*M* *_r_* = 452.75
Monoclinic, 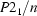

*a* = 12.872 (3) Å
*b* = 7.613 (2) Å
*c* = 20.695 (3) Å
β = 98.931 (3)°
*V* = 2003.4 (8) Å^3^
*Z* = 4
Mo *K*α radiation
μ = 0.66 mm^−1^
*T* = 298 K
0.18 × 0.17 × 0.15 mm

#### Data collection

Bruker SMART CCD diffractometer
Absorption correction: multi-scan *SADABS* (Sheldrick, 1996 ▶) *T*_min_ = 0.890, *T*_max_ = 0.907
13592 measured reflections
4368 independent reflections
3104 reflections with *I* > 2σ(*I*)
*R*_int_ = 0.036

#### Refinement

*R*[*F* ^2^ > 2σ(*F* ^2^)] = 0.042
*wR*(*F* ^2^) = 0.109
*S* = 1.05
4368 reflections
268 parameters
1 restraint
H atoms treated by a mixture of independent and constrained refinement
Δρ_max_ = 0.34 e Å^−3^
Δρ_min_ = −0.43 e Å^−3^

### 

Data collection: *SMART* (Bruker, 1998 ▶); cell refinement: *SAINT* (Bruker, 1998 ▶); data reduction: *SAINT*; program(s) used to solve structure: *SHELXS97* (Sheldrick, 2008 ▶); program(s) used to refine structure: *SHELXL97* (Sheldrick, 2008 ▶); molecular graphics: *SHELXTL* (Sheldrick, 2008 ▶); software used to prepare material for publication: *SHELXTL*.

## Supplementary Material

Crystal structure: contains datablock(s) global, I. DOI: 10.1107/S1600536811032703/lh5304sup1.cif

Structure factors: contains datablock(s) I. DOI: 10.1107/S1600536811032703/lh5304Isup2.hkl

Additional supplementary materials:  crystallographic information; 3D view; checkCIF report

## Figures and Tables

**Table 1:** Hydrogen-bond geometry (Å, °)

*D*—H⋯*A*	*D*—H	H⋯*A*	*D*⋯*A*	*D*—H⋯*A*
O4—H4⋯N2^i^	0.85 (1)	1.99 (1)	2.839 (3)	178 (4)

Symmetry code: (i) 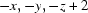.

**Fig. 1.**
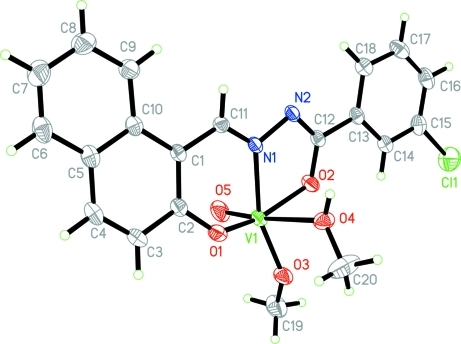
The molecular structure of the title compound with displacement ellipsoids are drawn at the 30% probability level.

**Fig. 2.**
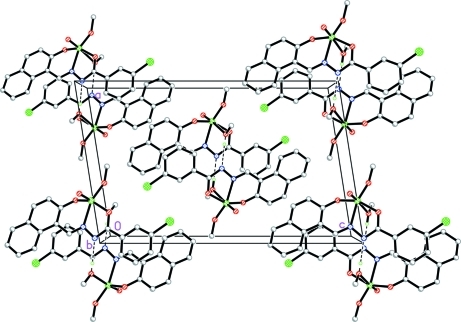
Part of the crystal structure with hydrogen bonds shown as dashed lines.

## supplementary crystallographic information

### Comment

Hydrazone compounds and their oxovanadium complexes have received much attention due to their structural and biological properties (Seena *et al.*, 2008; Bastos *et al.*, 2008; Sarkar & Pal, 2008; Nica *et al.*, 2007). Recently, the author has reported a oxovanadium complex derived from N'-(5-bromo-2-hydroxybenzylidene)-2-chlorobenzohydrazide (Wang, 2011). In this paper, the crystal structure of the title compound is reported.

The V^V^ ion in the title complex, Fig. 1, is six-coordinated by the phenolic O, imine N, and enolic O atoms of the hydrazone ligand, by one oxo O atom, and by two O atoms respectively from a methanol molecule and a methanolate ligand, forming a distorted octahedral geometry. The dihedral angle between the benzene ring and the naphthylene ring system of the hydrazone ligand is 6.4 (3)°. The deviation of the V atom from the plane defined by the three donor atoms of the hydrazone ligand and the methoxy O atom towards the oxo O atom is 0.323 (2) Å. The bond lengths and angles at the V^V^ ion are comparable with those observed in similar oxovanadium(V) complexes (Kurup *et al.*, 2010; Rajak *et al.*, 2000; Grüning *et al.*, 1999; Mondal *et al.*, 2009). In the crystal, pairs of intermolecular O—H···N hydrogen bonds form centrosymmetric dimers (Fig. 2).

### Experimental

2-Hydroxy-1-naphthaldehyde (1 mmol, 0.17 g), 3-chlorobenzohydrazide 1 mmol, 0.17 g), and VO(acac)_2_ (1 mmol, 0.26 g) were mixed in methanol (30 ml). The mixture was boiled under reflux for 2 h, then cooled to room temperature. Brown block-like single crystals, suitable for X-ray diffraction, were formed after slow evaporation of the solution in air for a few days.

### Refinement

Atom H4 was located from a difference Fourier map and refined isotropically. The O4—H4 distance was restrained to 0.85 (1) Å. The remaining hydrogen atoms were placed in geometrically idealized positions and constrained to ride on their parent atoms, with C—H distances of 0.93–0.96 Å, and with *U*_iso_(H) set at 1.2*U*_eq_(C) and 1.5*U*_eq_(C_methyl_).

### Crystal data

**Table:**

[V(C_18_H_11_ClN_2_O_2_)(CH_3_O)O(CH_4_O)]	*F*(000) = 928
*M**_r_* = 452.75	*D*_x_ = 1.501 Mg m^−^^3^
Monoclinic, *P*2_1_/*n*	Mo *K*α radiation, λ = 0.71073 Å
Hall symbol: -P 2yn	Cell parameters from 3491 reflections
*a* = 12.872 (3) Å	θ = 2.8–26.0°
*b* = 7.613 (2) Å	µ = 0.66 mm^−^^1^
*c* = 20.695 (3) Å	*T* = 298 K
β = 98.931 (3)°	Block, brown
*V* = 2003.4 (8) Å^3^	0.18 × 0.17 × 0.15 mm
*Z* = 4	

### Data collection

**Table:**

Bruker SMART CCD diffractometer	4368 independent reflections
Radiation source: fine-focus sealed tube	3104 reflections with *I* > 2σ(*I*)
graphite	*R*_int_ = 0.036
ω scan	θ_max_ = 27.0°, θ_min_ = 2.9°
Absorption correction: multi-scan *SADABS* (Sheldrick, 1996)	*h* = −14→16
*T*_min_ = 0.890, *T*_max_ = 0.907	*k* = −9→9
13592 measured reflections	*l* = −26→26

### Refinement

**Table:**

Refinement on *F*^2^	Primary atom site location: structure-invariant direct methods
Least-squares matrix: full	Secondary atom site location: difference Fourier map
*R*[*F*^2^ > 2σ(*F*^2^)] = 0.042	Hydrogen site location: inferred from neighbouring sites
*wR*(*F*^2^) = 0.109	H atoms treated by a mixture of independent and constrained refinement
*S* = 1.05	*w* = 1/[σ^2^(*F*_o_^2^) + (0.042*P*)^2^ + 0.9229*P*] where *P* = (*F*_o_^2^ + 2*F*_c_^2^)/3
4368 reflections	(Δ/σ)_max_ < 0.001
268 parameters	Δρ_max_ = 0.34 e Å^−^^3^
1 restraint	Δρ_min_ = −0.43 e Å^−^^3^

### Special details

**Table:**

Geometry. All esds (except the esd in the dihedral angle between two l.s. planes) are estimated using the full covariance matrix. The cell esds are taken into account individually in the estimation of esds in distances, angles and torsion angles; correlations between esds in cell parameters are only used when they are defined by crystal symmetry. An approximate (isotropic) treatment of cell esds is used for estimating esds involving l.s. planes.
Refinement. Refinement of F^2^ against ALL reflections. The weighted R-factor wR and goodness of fit S are based on F^2^, conventional R-factors R are based on F, with F set to zero for negative F^2^. The threshold expression of F^2^ > 2sigma(F^2^) is used only for calculating R-factors(gt) etc. and is not relevant to the choice of reflections for refinement. R-factors based on F^2^ are statistically about twice as large as those based on F, and R- factors based on ALL data will be even larger.

### Fractional atomic coordinates and isotropic or equivalent isotropic displacement parameters (Å^2^)

**Table:**

	*x*	*y*	*z*	*U*_iso_*/*U*_eq_	
V1	0.26454 (3)	0.08667 (6)	0.99698 (2)	0.03892 (14)	
Cl1	0.15025 (8)	0.45711 (15)	1.28761 (4)	0.0857 (3)	
N1	0.10581 (15)	0.1236 (2)	0.95334 (9)	0.0317 (4)	
N2	0.04044 (15)	0.2021 (3)	0.99338 (9)	0.0338 (5)	
O1	0.26311 (14)	−0.0734 (3)	0.92934 (8)	0.0472 (5)	
O2	0.19838 (13)	0.2083 (2)	1.06222 (8)	0.0417 (4)	
O3	0.37183 (13)	0.0107 (3)	1.05311 (8)	0.0467 (5)	
O4	0.17893 (14)	−0.1470 (3)	1.04327 (9)	0.0469 (5)	
O5	0.31027 (15)	0.2518 (3)	0.96509 (10)	0.0601 (5)	
C1	0.11838 (18)	0.0029 (3)	0.84704 (11)	0.0328 (5)	
C2	0.21637 (19)	−0.0746 (3)	0.86749 (12)	0.0367 (6)	
C3	0.2684 (2)	−0.1682 (4)	0.82235 (13)	0.0438 (6)	
H3	0.3329	−0.2215	0.8365	0.053*	
C4	0.2240 (2)	−0.1799 (4)	0.75880 (13)	0.0451 (7)	
H4A	0.2592	−0.2412	0.7299	0.054*	
C5	0.1258 (2)	−0.1019 (3)	0.73489 (12)	0.0418 (6)	
C6	0.0817 (2)	−0.1125 (4)	0.66811 (13)	0.0547 (8)	
H6	0.1183	−0.1709	0.6392	0.066*	
C7	−0.0130 (3)	−0.0392 (5)	0.64528 (15)	0.0657 (9)	
H7	−0.0405	−0.0465	0.6011	0.079*	
C8	−0.0688 (2)	0.0469 (4)	0.68830 (15)	0.0626 (9)	
H8	−0.1343	0.0951	0.6727	0.075*	
C9	−0.0285 (2)	0.0615 (4)	0.75334 (13)	0.0476 (7)	
H9	−0.0670	0.1207	0.7810	0.057*	
C10	0.07055 (19)	−0.0116 (3)	0.77942 (11)	0.0360 (6)	
C11	0.06312 (18)	0.0867 (3)	0.89423 (11)	0.0331 (5)	
H11	−0.0072	0.1158	0.8813	0.040*	
C12	0.09845 (18)	0.2435 (3)	1.04907 (11)	0.0332 (5)	
C13	0.05144 (19)	0.3322 (3)	1.10122 (11)	0.0345 (5)	
C14	0.1116 (2)	0.3473 (3)	1.16256 (12)	0.0403 (6)	
H14	0.1790	0.2999	1.1703	0.048*	
C15	0.0718 (2)	0.4319 (4)	1.21188 (13)	0.0485 (7)	
C16	−0.0294 (2)	0.4999 (4)	1.20174 (14)	0.0525 (7)	
H16	−0.0565	0.5546	1.2357	0.063*	
C17	−0.0890 (2)	0.4853 (4)	1.14100 (14)	0.0506 (7)	
H17	−0.1569	0.5308	1.1339	0.061*	
C18	−0.0496 (2)	0.4041 (3)	1.09024 (13)	0.0424 (6)	
H18	−0.0902	0.3972	1.0490	0.051*	
C19	0.4735 (2)	0.0765 (5)	1.07551 (15)	0.0683 (10)	
H19A	0.4918	0.1617	1.0450	0.102*	
H19B	0.5232	−0.0183	1.0793	0.102*	
H19C	0.4746	0.1308	1.1175	0.102*	
C20	0.2289 (3)	−0.3005 (5)	1.0690 (2)	0.0865 (12)	
H20A	0.2443	−0.3737	1.0340	0.130*	
H20B	0.1835	−0.3626	1.0939	0.130*	
H20C	0.2932	−0.2706	1.0970	0.130*	
H4	0.1136 (9)	−0.162 (5)	1.0318 (16)	0.086 (12)*	

### Atomic displacement parameters (Å^2^)

**Table:**

	*U*^11^	*U*^22^	*U*^33^	*U*^12^	*U*^13^	*U*^23^
V1	0.0281 (2)	0.0508 (3)	0.0397 (2)	0.0009 (2)	0.01096 (17)	0.0007 (2)
Cl1	0.0987 (7)	0.1131 (8)	0.0431 (4)	0.0125 (6)	0.0045 (4)	−0.0194 (5)
N1	0.0312 (10)	0.0324 (11)	0.0345 (10)	0.0022 (9)	0.0144 (8)	0.0005 (8)
N2	0.0319 (11)	0.0376 (12)	0.0348 (10)	0.0026 (9)	0.0140 (9)	−0.0003 (9)
O1	0.0402 (10)	0.0614 (12)	0.0407 (10)	0.0165 (9)	0.0080 (8)	−0.0048 (9)
O2	0.0298 (9)	0.0546 (12)	0.0417 (9)	−0.0012 (8)	0.0091 (7)	−0.0083 (8)
O3	0.0282 (9)	0.0654 (13)	0.0460 (10)	−0.0008 (9)	0.0045 (8)	−0.0069 (9)
O4	0.0343 (11)	0.0462 (11)	0.0585 (12)	−0.0021 (9)	0.0016 (9)	0.0122 (9)
O5	0.0502 (12)	0.0660 (14)	0.0703 (13)	−0.0063 (11)	0.0291 (10)	0.0096 (11)
C1	0.0322 (13)	0.0325 (13)	0.0359 (12)	−0.0026 (11)	0.0124 (10)	−0.0003 (10)
C2	0.0361 (13)	0.0364 (14)	0.0400 (13)	−0.0006 (11)	0.0131 (11)	−0.0003 (11)
C3	0.0336 (14)	0.0470 (16)	0.0535 (16)	0.0038 (12)	0.0153 (12)	−0.0045 (13)
C4	0.0449 (16)	0.0456 (17)	0.0495 (16)	−0.0048 (13)	0.0225 (13)	−0.0116 (13)
C5	0.0441 (15)	0.0394 (15)	0.0447 (14)	−0.0127 (12)	0.0154 (12)	−0.0065 (12)
C6	0.0572 (19)	0.064 (2)	0.0453 (15)	−0.0155 (16)	0.0163 (14)	−0.0188 (14)
C7	0.061 (2)	0.087 (3)	0.0462 (17)	−0.0128 (19)	−0.0015 (15)	−0.0195 (17)
C8	0.0508 (18)	0.079 (2)	0.0538 (18)	0.0010 (16)	−0.0059 (15)	−0.0124 (16)
C9	0.0415 (15)	0.0559 (18)	0.0446 (15)	0.0002 (13)	0.0039 (12)	−0.0104 (13)
C10	0.0365 (14)	0.0342 (14)	0.0390 (13)	−0.0078 (11)	0.0113 (11)	−0.0027 (11)
C11	0.0285 (12)	0.0347 (13)	0.0372 (12)	−0.0003 (11)	0.0082 (10)	0.0046 (11)
C12	0.0323 (13)	0.0315 (13)	0.0382 (12)	−0.0042 (11)	0.0129 (10)	0.0017 (10)
C13	0.0358 (13)	0.0300 (13)	0.0406 (13)	−0.0053 (11)	0.0142 (11)	−0.0005 (10)
C14	0.0394 (14)	0.0429 (15)	0.0408 (14)	−0.0016 (12)	0.0134 (11)	−0.0005 (11)
C15	0.0587 (18)	0.0502 (18)	0.0392 (14)	−0.0060 (15)	0.0163 (13)	−0.0045 (12)
C16	0.063 (2)	0.0482 (18)	0.0529 (17)	−0.0029 (15)	0.0306 (15)	−0.0096 (14)
C17	0.0446 (16)	0.0410 (16)	0.071 (2)	0.0002 (13)	0.0229 (15)	−0.0087 (14)
C18	0.0435 (15)	0.0377 (15)	0.0474 (15)	−0.0021 (12)	0.0111 (12)	−0.0042 (12)
C19	0.0375 (16)	0.108 (3)	0.0586 (19)	−0.0146 (17)	0.0047 (14)	−0.0152 (19)
C20	0.070 (2)	0.065 (2)	0.111 (3)	−0.0051 (19)	−0.026 (2)	0.032 (2)

### Geometric parameters (Å, °)

**Table:**

V1—O5	1.576 (2)	C6—H6	0.9300
V1—O3	1.7587 (18)	C7—C8	1.392 (4)
V1—O1	1.8539 (18)	C7—H7	0.9300
V1—O2	1.9398 (17)	C8—C9	1.370 (4)
V1—N1	2.120 (2)	C8—H8	0.9300
V1—O4	2.371 (2)	C9—C10	1.419 (4)
Cl1—C15	1.738 (3)	C9—H9	0.9300
N1—C11	1.292 (3)	C11—H11	0.9300
N1—N2	1.403 (3)	C12—C13	1.480 (3)
N2—C12	1.311 (3)	C13—C14	1.385 (3)
O1—C2	1.327 (3)	C13—C18	1.397 (4)
O2—C12	1.301 (3)	C14—C15	1.371 (4)
O3—C19	1.410 (3)	C14—H14	0.9300
O4—C20	1.400 (4)	C15—C16	1.387 (4)
O4—H4	0.845 (10)	C16—C17	1.372 (4)
C1—C2	1.397 (3)	C16—H16	0.9300
C1—C10	1.443 (3)	C17—C18	1.382 (4)
C1—C11	1.443 (3)	C17—H17	0.9300
C2—C3	1.423 (3)	C18—H18	0.9300
C3—C4	1.353 (4)	C19—H19A	0.9600
C3—H3	0.9300	C19—H19B	0.9600
C4—C5	1.415 (4)	C19—H19C	0.9600
C4—H4A	0.9300	C20—H20A	0.9600
C5—C6	1.413 (4)	C20—H20B	0.9600
C5—C10	1.425 (3)	C20—H20C	0.9600
C6—C7	1.357 (4)		
			
O5—V1—O3	103.46 (10)	C9—C8—C7	120.8 (3)
O5—V1—O1	99.70 (10)	C9—C8—H8	119.6
O3—V1—O1	101.38 (8)	C7—C8—H8	119.6
O5—V1—O2	98.26 (9)	C8—C9—C10	121.5 (3)
O3—V1—O2	94.70 (8)	C8—C9—H9	119.2
O1—V1—O2	152.31 (8)	C10—C9—H9	119.2
O5—V1—N1	96.54 (9)	C9—C10—C5	116.9 (2)
O3—V1—N1	158.57 (8)	C9—C10—C1	124.2 (2)
O1—V1—N1	82.51 (8)	C5—C10—C1	118.9 (2)
O2—V1—N1	74.64 (7)	N1—C11—C1	123.7 (2)
O5—V1—O4	174.17 (9)	N1—C11—H11	118.1
O3—V1—O4	81.55 (8)	C1—C11—H11	118.1
O1—V1—O4	82.03 (8)	O2—C12—N2	123.0 (2)
O2—V1—O4	78.18 (8)	O2—C12—C13	116.4 (2)
N1—V1—O4	78.11 (7)	N2—C12—C13	120.6 (2)
C11—N1—N2	116.49 (19)	C14—C13—C18	119.4 (2)
C11—N1—V1	127.92 (16)	C14—C13—C12	118.2 (2)
N2—N1—V1	115.56 (14)	C18—C13—C12	122.4 (2)
C12—N2—N1	108.04 (19)	C15—C14—C13	120.1 (3)
C2—O1—V1	133.04 (16)	C15—C14—H14	120.0
C12—O2—V1	118.64 (15)	C13—C14—H14	120.0
C19—O3—V1	133.9 (2)	C14—C15—C16	120.8 (3)
C20—O4—V1	124.4 (2)	C14—C15—Cl1	119.5 (2)
C20—O4—H4	112 (3)	C16—C15—Cl1	119.7 (2)
V1—O4—H4	119 (2)	C17—C16—C15	119.2 (3)
C2—C1—C10	119.4 (2)	C17—C16—H16	120.4
C2—C1—C11	119.9 (2)	C15—C16—H16	120.4
C10—C1—C11	120.6 (2)	C16—C17—C18	120.9 (3)
O1—C2—C1	122.8 (2)	C16—C17—H17	119.5
O1—C2—C3	116.6 (2)	C18—C17—H17	119.5
C1—C2—C3	120.5 (2)	C17—C18—C13	119.6 (3)
C4—C3—C2	120.1 (2)	C17—C18—H18	120.2
C4—C3—H3	120.0	C13—C18—H18	120.2
C2—C3—H3	120.0	O3—C19—H19A	109.5
C3—C4—C5	122.1 (2)	O3—C19—H19B	109.5
C3—C4—H4A	118.9	H19A—C19—H19B	109.5
C5—C4—H4A	118.9	O3—C19—H19C	109.5
C6—C5—C4	121.3 (2)	H19A—C19—H19C	109.5
C6—C5—C10	119.7 (3)	H19B—C19—H19C	109.5
C4—C5—C10	119.0 (2)	O4—C20—H20A	109.5
C7—C6—C5	121.3 (3)	O4—C20—H20B	109.5
C7—C6—H6	119.3	H20A—C20—H20B	109.5
C5—C6—H6	119.3	O4—C20—H20C	109.5
C6—C7—C8	119.7 (3)	H20A—C20—H20C	109.5
C6—C7—H7	120.1	H20B—C20—H20C	109.5
C8—C7—H7	120.1		

### Hydrogen-bond geometry (Å, °)

**Table:**

*D*—H···*A*	*D*—H	H···*A*	*D*···*A*	*D*—H···*A*
O4—H4···N2^i^	0.85 (1)	1.99 (1)	2.839 (3)	178 (4)

Symmetry codes: (i) −*x*, −*y*, −*z*+2.
